# Evaluation of Antibiotic Resistance Mechanisms in Gram-Negative Bacteria

**DOI:** 10.3390/antibiotics12111590

**Published:** 2023-11-03

**Authors:** Anusha Gauba, Khondaker Miraz Rahman

**Affiliations:** Institute of Pharmaceutical Science, King’s College London, 150 Stamford Street, London SE1 9NH, UK; anusha.gauba@kcl.ac.uk

**Keywords:** antibiotic resistance, Gram-negative, pathogens, antimicrobial, ESKAPE, multidrug resistance (MDR), resistance mechanisms, *E. coli*, *A. baumannii*, *P. aeruginosa*, *K. pneunomiae*

## Abstract

Multidrug-resistant Gram-negative bacterial infections are exponentially increasing, posing one of the most urgent global healthcare and economic threats. Due to the lack of new therapies, the World Health Organization classified these bacterial species as priority pathogens in 2017, known as ESKAPE pathogens. This classification emphasizes the need for urgent research and development of novel targeted therapies. The majority of these priority pathogens are Gram-negative species, which possess a structurally dynamic cell envelope enabling them to resist multiple antibiotics, thereby leading to increased mortality rates. Despite 6 years having passed since the WHO classification, the progress in generating new treatment ideas has not been sufficient, and antimicrobial resistance continues to escalate, acting as a global ticking time bomb. Numerous efforts and strategies have been employed to combat the rising levels of antibiotic resistance by targeting specific resistance mechanisms. These mechanisms include antibiotic inactivating/modifying enzymes, outer membrane porin remodelling, enhanced efflux pump action, and alteration of antibiotic target sites. Some strategies have demonstrated clinical promise, such as the utilization of beta-lactamase inhibitors as antibiotic adjuvants, as well as recent advancements in machine-based learning employing artificial intelligence to facilitate the production of novel narrow-spectrum antibiotics. However, further research into an enhanced understanding of the precise mechanisms by which antibiotic resistance occurs, specifically tailored to each bacterial species, could pave the way for exploring narrow-spectrum targeted therapies. This review aims to introduce the key features of Gram-negative bacteria and their current treatment approaches, summarizing the major antibiotic resistance mechanisms with a focus on *Escherichia coli*, *Acinetobacter baumannii*, *Pseudomonas aeruginosa*, and *Klebsiella pneumoniae*. Additionally, potential directions for alternative therapies will be discussed, along with their relative modes of action, providing a future perspective and insight into the discipline of antimicrobial resistance.

## 1. Introduction

Gram-negative bacteria are classified by their multi-layered macromolecular structure known as the cell envelope, consisting of three components (beginning from the outside of the cell): the outer (periplasmic) membrane (OM), the peptidoglycan layer, and the inner (cytoplasmic) membrane (IM). The OM and IM together surround the periplasm, an aqueous chamber within the two membranes that contains the peptidoglycan layer. Each component is vital for bacterial viability, having different roles and functions in cell envelope integrity [1].

**Figure 1:**
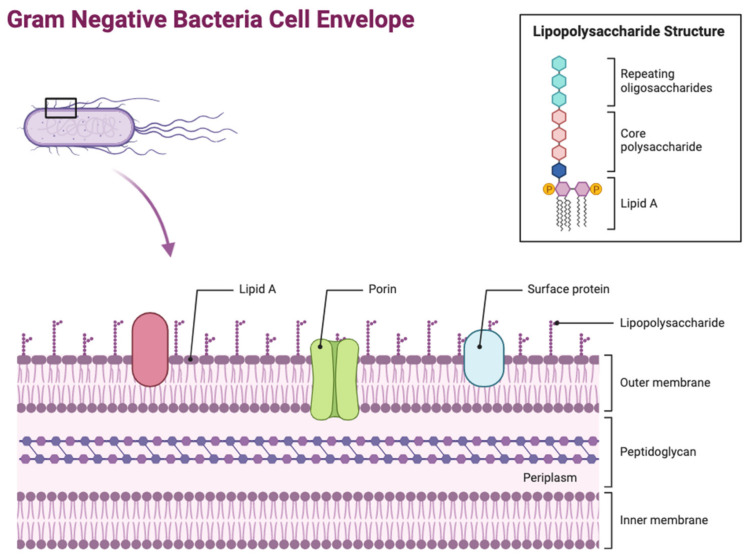
Architecture of the Gram-negative bacterial cell envelope. Figure 1 depicts the structure of the Gram-negative bacterial cell envelope, showing its distinct layers including the peptidoglycan layer located in the periplasm. Several LPS molecules can be seen attached to the OM surface, alongside the presence of major OM proteins such as porins. Lipid A is specifically labelled as part of the LPS due to its significant role in virulence and resistance [1] (figure created using BioRender, https://www.biorender.com/).

The OM is one of the most distinct features of Gram-negative bacteria, differentiating it from Gram-positive bacteria. Its main function involves protecting the bacteria cell from its external environment, acting as a permeability barrier preventing entry of harmful compounds. Its asymmetric structure is overall classified as a lipid bilayer, consisting of two leaflets (Figure 1). The outer leaflet is composed of lipopolysaccharides (LPSs) and the inner leaflet is composed of phospholipids, with the OM being dispersed with several integral proteins being grouped into two categories: OM proteins (OMPs) known as β-barrel proteins, and lipoproteins [2]. The lipoproteins are found in the OM inner leaflet, anchored to the membrane by their lipid moieties that are linked to an amino-terminal cysteine residue. Different types of Gram-negative Bacteria can produce several varied lipoproteins, such as *E. coli*, which produces approximately 80 different lipoproteins. These lipoproteins have several essential roles including cell virulence, peptidoglycan remodelling and synthesis, cell architecture, responses to cell stress, cell division, and OM biogenesis [3]. However, the most predominant proteins found in the OM are OM proteins (OMPs) in the form of β-barrels. The β-barrel structure comprises an asymmetric barrel-like structure within the membrane where the periplasmic side has short loops within each strand and the extracellular side has bigger, more extended loops. The majority of these proteins all have an even number of β-strands, which, when incorporated together, form in an antiparallel arrangement. Due to their location being on the exterior of the bacterium, β-barrel OMPs are the initial point of contact between the bacterium and the extracellular environment. This means that they have a wide range of functions, including being responsible for modulating the entry of hydrophilic antibiotics and nutrients into the bacterium, as well as stabilising the bacterial cell envelope. Furthermore, they also act as adhesins in bacterial pathogenesis, as well as allowing the influx of siderophore receptors, lipases, and proteases [4]. LPS is a key glycolipid molecule involved in maintaining the structure and permeability of Gram-negative bacteria, alongside playing a key role in bacteria virulence by regulating the host’s immune response [5].

The peptidoglycan layer is a key aspect of the bacterial cell envelope, composed of linear glycan strands forming a scaffold-like complex around the bacterial IM. The glycan strands are formed from alternate residues of N-acetylmuramic acid (Mur*N*Ac) and ß-1,4-linked N-acetylglucosamine (Glc*N*Ac) and cross-linked via covalent interactions by small peptides to create a mesh-like structure known as the peptidoglycan sacculus [6]. In Gram-negative bacteria, this layer is much thinner compared to Gram-positive bacteria, and these peptides interact with each other via d-alanine^4^ and meso-diaminopimelic acid^3^ (mDAP^3^), or two mDAP^3^ residues of consecutive peptides from neighbouring glycan polymers [7].

The IM is a symmetric membrane formed of a phospholipid bilayer where several proteins responsible for membrane-associated functions, such as lipid and protein biosynthesis and secretion, are found. It is the key region for DNA anchoring and is heavily involved in the separation of sister chromosomes [8]. Most of the proteins located in the IM have the structure of α-helices with membrane spanning regions [9]. All proteins and elements of the cell envelope are produced either in the inner surface of the IM or synthesized in the cytoplasm before being translocated to their designated region [2].

### Antimicrobial Resistance and Multidrug Resistance

**Figure 2:**
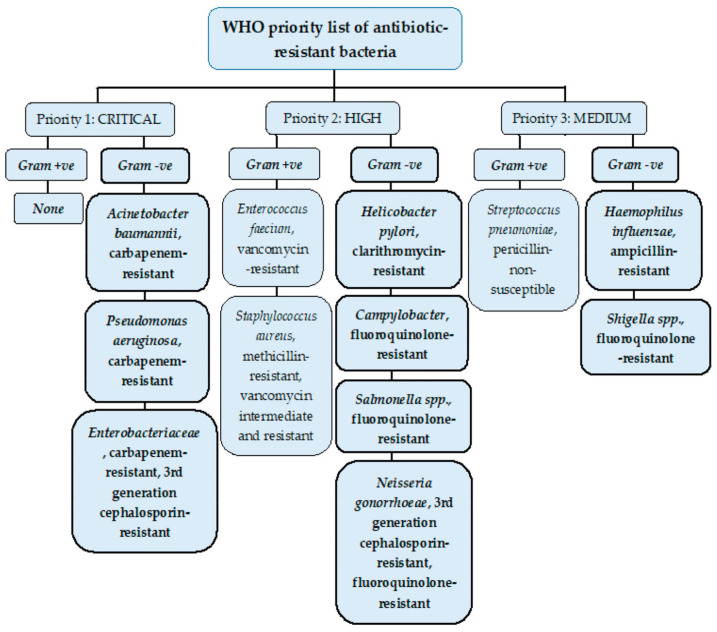
WHO priority list of pathogens classified from highest to lowest priority based on their antibiotic resistance levels. Many of the bacteria shown in the WHO priority list belong to ESKAPE pathogens, a group of serious life-threatening pathogens worldwide that are classified as MDR pathogens. These bacteria are *Enterococcus faecium*, *Staphylococcus aureus*, *Klebsiella pneumoniae*, *Acinetobacter baumannii*, *Pseudomonas aeruginosa*, *and Enterobacter* bacterium types. All the ESKAPE bacteria are Gram-negative apart from Enterococcus faecium, highlighting the need to especially tackle MDR Gram-negative bacteria [13]. Figure adapted from [8].

Antimicrobial resistance (AMR) is a rapidly growing global phenomenon, emerging as one of the top ten global threats declared by the World Health Organization (WHO). It occurs when bacteria, fungi, viruses, and parasites evolve over time, acquiring new mechanisms to evade antimicrobial treatment. One of the major concerns of AMR includes antibiotic resistance, which refers to the evolution of bacteria and their resistance to antibiotic drugs [10]. When bacterial pathogens have become resistant or insensitive to high doses of several classes of antibiotics, they are referred to as multidrug-resistant (MDR) [11]. The urgent requirement for new antibiotics to treat MDR bacteria is imperative, with some strains acquiring resistance to nearly all types of antibiotics [10]. A list by the WHO was published in 2017 that identified a list of ‘priority pathogens’, which were and are increasingly difficult to treat, many of these being Gram-negative bacteria species due to their unique cell envelope structure. This was to encourage research and development teams to respond to the urgent need for new drugs, especially for MDR bacteria including *Acinetobacter*, *Pseudomonas*, and *Enterobacteriaceae*. These Gram-negative bacteria families can cause fatal bloodstream infections and pneumonia and are categorised of the highest urgency in this list (See Figure 2) [8,12].

The statistics of MDR bacterial infections are continuously rising, with several economic effects as well as effects on healthcare. Reports have estimated that EUR 1.5 billion would be spent per year in Europe of their economy to match the mortality rate as a result of MDR infections [14]. Studies by O’Neill in 2014 and 2015 focus on the potential global effects and burdens we may face if the increasing AMR and MDR bacteria were to be ignored. It was projected that if in 50 years’ time if we were to continue without any improvements made to policies and research and drug development, world GDP could decrease by USD 55 trillion, with 150 million premature deaths. It was also determined that if action was to be taken now rather than in 10 years’ time, USD 65 trillion could be saved between now and 2050. However, this projection is only based on 3/7 of the most resistant bacteria posing the highest threat according to the World Health Organization (WHO): *Klebsiella pneumoniae*, *E. coli*, *and Staphylococcus aureus.* When all seven bacteria are considered, the threat and implications are much higher, with projected statistics for only three bacteria already being severely detrimental. Secondary effects of AMR were also researched, with the impact on advances made in medicine investigated as well as the impact on surgical and non-surgical treatments. If AMR is not tackled soon, treatments like chemotherapy, which is crucial to cancer treatment, may not be able to be as easily taken. This is due to the immunosuppressing side effects of chemotherapy, making patients more susceptible to bacterial infections with AMR causing a lack of effective treatment. Furthermore, surgeries with high chances of bacterial infection development, such as bowel surgery, will not be routine surgeries, and other general surgeries such as caesarean surgeries will also not be routine, increasing the infant mortality rate [15,16].

## 2. Antibiotic Resistance Mechanisms in Gram-Negative Bacteria

Gram-negative bacteria resistant to antibiotics listed above have been increasingly found in humans, with these bacteria being able to evade antimicrobial killing using a range of different mechanisms. Bacteria can already have intrinsic resistance against particular antibiotic classes, but most of these mechanisms are found on mobile genetic elements (MGEs) that are transferred between related bacteria [17]. Bacteria can adapt to new environments by evolving to escape the host immune defences in the presence of several selective pressures through genetic changes. This can be through the acquisition of different mutations, and sometimes by acquiring new genes from different bacteria, known as horizontal gene transfer (HGT). HGT allows bacteria to respond to their selective pressures, i.e., new antibiotics, much faster than bacterial mutations, by the singular transfer of substantial DNA sequences from one bacterium to another. This allows for changes to the bacterial genome at a much larger scale, allowing bacteria to obtain several virulence genes known as pathogenicity islands. HGT in bacteria occurs through three major mechanisms, transformation, transduction, and conjugation, with conjugation being the most common pathway [18,19]. HGT allows for bacteria to spread AMR genes producing ‘superbugs’, which harbour multiple antibiotic resistance genes on plasmids and are resistant to almost all antibiotics [20].

These mechanisms of resistance are understood to affect all current antibiotics, with the exception of some new drugs that are considered for use when first-line drugs are not effective. Antibiotic resistance mechanisms can be characterized into four main groups:

(1) Drug inactivation;
(2) Limiting drug uptake;
(3) Altering drug target;
(4) High levels of drug efflux.

**Figure 3:**
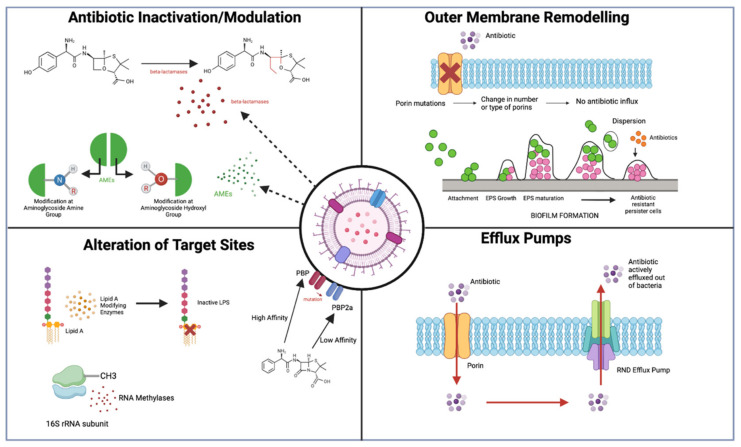
Overview of main discussed antibiotic resistance mechanisms in Gram-negative bacteria. Figure 3 outlines the 4 major resistance mechanisms discussed in this review for Gram-negative bacteria, including the activity of beta-lactamases and AMEs to inactivate/modify antibiotics; remodelling of the OM porins and biofilm formation; alteration of antibiotic target sites in the bacterium such as Lipid A, 16s rRNA, and PBPs; and increased efflux pump action to actively transport antibiotics out of the cell (figure created using BioRender), adapted from [12].

Gram-negative bacteria have been shown to be able to acquire all these mechanisms of resistance due to a variety of different proteins and pathways [21]. AMR can occur due to several mechanisms including direct inactivation or decreasing the intracellular concentration of the drug, and modulation or protection of the target site (Figure 3). The increasing mobilization of these genes encoding for resistance mechanisms to Gram-negative pathogens is presenting as an increasing challenge due to the absence of functional antibiotics. Our advancing understanding and knowledge regarding the molecular mechanisms behind antibiotic resistance should be considered when developing novel antibiotics, to produce new drugs to circumvent these mechanisms [22].

### 2.1. Antibiotic Inactivation/Modulation

Inactivation and alteration of antibiotics is one of the most common methods used by Gram-negative bacteria to evade their action, usually by the assembly of enzymes that result in the irreversible destruction or neutralization of antibiotics. The mechanism of action of these enzymes can include impairing the enzyme active site, preventing binding, e.g., the hydrolytic cleavage action of β-lactamases on the β-lactam ring of β-lactam antibiotics. Another mode of action includes the covalent alteration of important structural antibiotic features, preventing its interaction with the drug-target site on Gram-negative bacteria, e.g., the alteration of hydroxyl/amino groups on antibiotics by aminoglycoside-modifying enzymes (AMEs) [12]. Antibiotics function by binding to their bacterial target with a high affinity, allowing their influx and antimicrobial killing mechanisms. When modifications are made to the bacterial targets, efficiency of antibiotic binding decreases, thus reducing their inhibitory effect. There are several components of Gram-negative bacteria that are targets for antibiotic drugs, as well as many targets that are able to be altered, enabling antibiotic resistance. Some of these targets include penicillin-binding proteins (PBPs) and Lipid A-modifying enzymes. The number or structure of PBPs can be altered, affecting the binding ability of antibiotics to their target, or completely inhibit binding [21].

#### 2.1.1. β-Lactamase-Antibiotic Inactivation

β-lactamases are enzymes found in Gram-negative bacteria that function by hydrolysing the amine bond (-CO-NH structure) in the core four-membered β-lactam ring in β-lactam antibiotics. This renders the antibiotic ineffective, preventing their action, thus evading their antimicrobial killing. β-lactamases can be extended-spectrum (ESBLs) or narrow-spectrum, with ESBLs becoming a major concern due to their ability to hydrolyse several β-lactam antibiotics. Overall, these enzymes are classified in two major ways, the Ambler classification where they are divided into four main molecular classes, A, B, C, and D, based on their amino acid motifs, or the functional classification by Bush–Jacobi–Medeiros [23]. According to the Ambler classification, classes A, C, and D have a common mechanism by which hydrolysis of the substrate occurs through the production of an acyl enzyme through serine acting as an active site. Class B β-lactamases (known as metallo-β-lactamases) are classified as metalloenzymes that use a minimum of one zinc as an active site to help promote hydrolysis of the β-lactam ring. 

According to the functional classification, these enzymes are divided into three groups (groups 1–3), based on the effects of β-lactamase inhibitors and breakdown of β-lactam substrates [24]. Group 1 is cephalosporinases, corresponding to molecular class C, and are found on the chromosomes belonging to many bacteria of Enterobacteriaceae. These enzymes are active against cephalosporins more than other β-lactams such as benzylpenicillins and are usually resistant to clavulanic acid, but active against cefoxitin. Group 2 is named serine β-lactamases and corresponds to molecular classes A and D and constitutes most of the β-lactamases. These enzymes have a higher efficiency when hydrolysing penicillin derivatives and benzylpenicillins rather than cephalosporins, carbapenems, and monobactams, where their hydrolysis is significantly lower. Group 3 is metallo-β-lactamases, corresponding to class C. This class has a low hydrolytic capability against monobactams and cannot be inhibited by clavulanic acid and is instead rendered ineffective when metal ion chelators are used against it such as EDTA [23].

#### 2.1.2. Aminoglycoside-Modifying Enzymes (AMEs)—Antibiotic Modification

AMEs are a key mechanism by which Gram-negative bacteria become MDR through antibiotic modification. They are crucial enzymes that catalyse the chemical modification of aminoglycoside antibiotics, resulting in their inactivity. This occurs at their -OH or -NH2 groups on the 2-deoxystreptamine nucleus or sugar groups and can be one of acetyltransferases (AACs), phosphotransferases (APHs), or nucleotidyltransferases (ANTs), each having a different mechanism. AACs function by catalysing the acetylation of -NH2 groups with the use of acetyl coenzyme A acting as a donor substrate to the acceptor molecule. ANTs prevent the action of aminoglycosides by catalysing the transfer of an AMP molecule to the -OH group on the antibiotic from ATP, which acts as a donor substrate. APHs allow addition of a phosphate group to the aminoglycoside, changing the distribution of the drug charge, thus inhibiting its interaction with the ribosome. Overall, these enzymes disrupt the chemical structure of aminoglycosides, reducing their affinity for their target site or preventing ribosomal binding, rendering the drug ineffective [25].

### 2.2. Limiting Influx of Antibiotics

Another mechanism of antibiotic resistance employed by various MDR Gram-negative species involves limiting the influx of antibiotics into the cell to prevent their action. Many antibiotics must cross the OM to reach the contents of the cell to achieve their antimicrobial effects. Due to this, many Gram-negative bacteria have developed these mechanisms to limit entry of antibiotics into the cell by decreasing influx such as regulating OM permeability and increasing the rate of efflux. This mechanism is especially important in Gram-negative bacteria due to many antibiotic targets being present in the IM. Hydrophilic molecules such as β-lactams, fluroquinolones, and tetracyclines are impacted by OM permeability changes due to their vital use of porins to cross the OM and reach their relative target sites [26]. Furthermore, increasing efflux of antibiotics is equally as important in AMR, and is achieved by specialised transporter proteins known as efflux pumps. Unlike most mechanisms of resistance discussed, efflux pumps are often intrinsic, with encoding genes located on an operon with its expression modulated at transcription. Mutations in the promotor regions or regulatory proteins of the operon cause an overexpression of these efflux pumps, resulting in AMR [27]. 

#### OM Remodelling

OM remodelling is a vital antibiotic resistance process observed in various Gram-negative bacteria, decreasing the influx of antibiotics, resulting in their survival. Membrane remodelling is a process by which bacteria regulate or remove specific components of the membrane such as lipids and proteins to adapt to a new environment. During this process, new proteins such as porins can be integrated into the membrane as well as the degradation of present proteins. This mechanism has recently been shown to have a significant impact on AMR rates, with this bacterial feature urgently requiring further research and targeted treatment. The degradation of present damaged proteins occurs via the action of BepA, YcaL, and DegP, which are a collection of proteases that break down OM proteins. The insertion of new proteins to the OM begins in the cytoplasm where protein precursors of porins are produced before being translocated to the IM and periplasm [28]. The porin precursor proteins are then merged into the OM via the beta-barrel assembly machinery (BAM) complex, which is responsible for integrating proteins as completely folded structures into the OM [29]. Membrane remodelling is a major mechanism of AMR, preventing antibiotic influx at the OM surface by the addition/removal of proteins, ensuring drug site targets inside the bacterium are kept safe from antimicrobial killing. Due to the impermeable nature of the lipopolysaccharide (LPS)–phospholipid OM, antibiotics enter via porins, with the most abundant type of porin present being classified as ‘major porins’. Bacterial OM porins are key water-filled protein channels that regulate nutrient influx in bacteria, essential for cell survival, and are responsible for the influx of antibiotics. A collection of regulator proteins are responsible for regulating the genes that encode different porins at a transcriptional as well as a post-transcriptional level. Examples of these proteins include CpxR and OmpR, which are two-component signalling systems, and small noncoding RNA proteins such as micF, micA, and micC, all having different functions in membrane remodelling when modulating porin production in the presence of different external environmental stimuli. These two-component signalling systems, such as the EnvZ-OmpR regulatory system, are able to detect osmolarity changes in the external environment or the presence of different antimicrobial drugs. This allows ‘remodelling’ changes to be made in OM protein composition. Furthermore, the CpxR alongside other proteins found in the Cpx envelope can also detect external changes in osmolarity and antimicrobial drugs, which trigger OM remodelling. This system acts together with the small noncoding RNA protein micC, which increases in the presence of antimicrobial drugs, particularly β-lactams, initiating OM remodelling [28]. 

### 2.3. Modifications of Antibiotic Targets

#### 2.3.1. Lipid A Modifications

Modifications of drug target sites found in Gram-negative bacteria are a key process by which they become MDR, with an example of this being alterations of Lipid A. Lipopolysaccharides (LPS) are key molecules found in the OM of Gram-negative bacteria, increasing bacterium stability, and protecting the bacteria from the external environment. Furthermore, they are key molecules in Gram-negative bacteria virulence, initiating host immune responses by interacting with host signalling receptors. Gram-negative bacteria have developed enzymes called Lipid A-modifying enzymes through evolution, modifying the Lipid A region of LPS that allows these bacteria to evade host immune responses. Examples of types of modifications that can occur by lipid A-modifying enzymes include the addition of N-Ara4N and phosphoethanolamine (pETtN), which are positively charged sugar groups. Further modification methods include acylation, fatty acyl chain deacylation, and hydroxylation. When the positively charged sugar groups are added to Lipid A, the overall negative charge of the Lipid A decreases, which decreases its ability to bind to cationic antimicrobial peptides (CAMPs), reducing the electrostatic interactions. Antibiotics such as polymyxins are dependent on their electrostatic interactions with the bacteria OM to disrupt the bacterial membranes, so when these modifications to Lipid A occur, these antibiotics are not as efficient, allowing bacteria to evade their antimicrobial mechanisms. This allows Gram-negative pathogens to survive despite the presence of antibiotics, increasing bacterial viability [30].

#### 2.3.2. 16S Ribosomal RNA Methylation

Another drug target site that can be modified, resulting in antibiotic resistance, is 16S ribosomal RNA (rRNA). The methylation of 16S rRNA in Gram-negative pathogens has emerged as a novel resistance mechanism specific towards aminoglycoside antibiotics reported in *A. baumannii* and *P. aeruginosa*. This mechanism is performed by 16S rRNA methylases, with common enzymes known as RmtB and RmtA. Genes encoding for these enzymes are located on ICEs such as transposons found in transferable plasmids. This allows their rapid dissemination throughout bacterial species via HGT and has a significant responsibility in the spread of aminoglycoside resistance. These enzymes have also been reported to co-localise with ESBLS such as MBLs, producing MDR Gram-negative pathogen strains [31].

### 2.4. Increasing Efflux of Antibiotics

#### Efflux Pumps

Efflux pumps are vital proteins involved in antibiotic resistance and are found in all bacterial species. These complex transporter proteins are usually located in the IM and OM of Gram-negative bacteria, responsible for the extrusion of noxious substances from the cell wall such as antibiotics. This results in a lower antibiotic concentration inside the cell, meaning the bacteria can withstand high concentrations of antibiotics [32]. 

**Figure 4:**
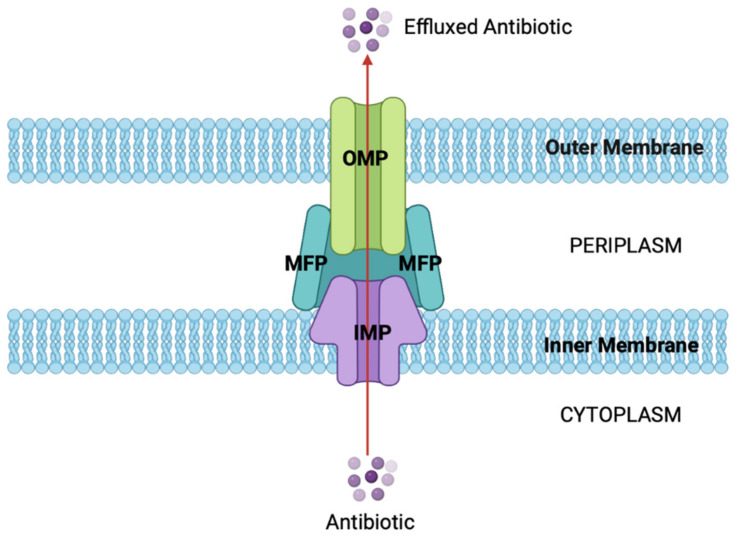
General structure and function of RND efflux pumps. The tripartite structure of RND efflux pumps, with the OMP shown in green, the MFPs shown in blue, and the IMP shown in purple. The pump spans the cell envelope, allowing antibiotics to be actively effluxed from the cytoplasm via PMF as an energy source. This decreases the intracellular antibiotic concentration, evading their antimicrobial effect (figure created using BioRender).

There have been six efflux pump families reported in the literature, classified as having the ability to extrude various antibiotic classes. These families include the small multidrug resistance (SMR) family, the major facilitator superfamily (MFS), the resistance–nodulation–division (RND) family, the ATP-binding cassette (ABC) superfamily, the multidrug and toxic compound extrusion (MATE) family, and the recently identified family known as the proteobacterial antimicrobial compound efflux (PACE) family. These active efflux systems play a crucial role in conferring resistance to a variety of chemically diverse antibiotics and antimicrobial agents, and instances of their occurrence are rapidly increasing in both clinical and environmental bacterial strains. Importantly, these systems are nearly ubiquitous across all bacterial kingdoms, contributing to antibiotic resistance through their shared common resistance mechanisms [33]. Overall, efflux pumps are divided into two groups based on their source of energy, either using ATP, which are known as primary efflux pumps, or the proton motive force (PMF), known as secondary efflux pumps [34]. The majority of Gram-negative efflux pumps belong to the resistance–nodulation–division (RND) family, and usually have a tripartite structure formed from an outer membrane protein (OMP), a membrane fusion protein (MFP), and an inner membrane protein (IMP) (Figure 4). The RND family are one of the four secondary efflux pump classes and are mostly organized as an operon. They have a broad involvement in AMR by increasing resistance to various antibiotics such as tetracyclines, fluoroquinolones, aminoglycosides, and penicillins [35]. 

This review will focus on antibiotic resistance mechanisms in *Escherichia* coli, *Acinetobacter baumannii*, *Klebsiella pneumoniae*, and *Pseudomonas aeruginosa*, with these Gram-negative bacteria being listed as the highest priority pathogens according to the WHO classification seen in Figure 2.

## 3. Key Multidrug-Resistant Gram-Negative Bacteria

### 3.1. Escherichia coli

*E. coli* is a common Gram-negative bacterium that belongs to the Enterobacteriacae family, causing several diseases such as urinary tract infections (UTIs), cystitis, bacteraemia, and pneumonia. There are multiple strains of this bacteria, resulting in a large scale of infections with different severities, ranging from mild gastrointestinal infections to severe disease, causing septic shock and renal failure. Furthermore, its role in nosocomial infections is significant, responsible for ventilator-associated pneumonia (VAP) and catheter-associated UTIs [36]. Despite *E. coli* being the causative pathogen for these diseases, many strains do not possess any virulence and are commensal strains belonging to our gut flora. These strains are vital for the vitamin K_2_ synthesis, important for blood clotting [37]. Antibiotics used against *E. coli* include mainly fluoroquinolones, such as ciprofloxacin, aminoglycosides, and macrolides such as azithromycin [36]. Data have shown that ciprofloxacin, an antibiotic used to treat *E. coli* infections, is one of the main antibiotics that have been inappropriately prescribed, resulting in the present highest resistance rates to *E. coli* worldwide [38]. Furthermore, a study by Zhang and colleagues in 2015 investigating links between epidemiology and antibiotic resistance rates in *E. coli* showed that phenotypes of various diarrheagenic virulence levels present with some correlation, explained by patients that presented with diarrhoea and had a significant history of antibiotic overuse before their symptom onset. This was suggested to be due to antibiotics affecting the gut microbiome, encouraging the growth of pathogens resistant to medication [39]. Over the last two decades, there has been a significant spread of *E. coli* strains resistant to several antibiotics, including β-lactams, quinolones, and aminoglycosides as well as last-resort drugs such as carbapenems and polymyxins [38]. The following sections will describe the various resistance mechanisms produced by this Gram-negative bacillus to their relative antibiotics. 

#### 3.1.1. β-Lactamases

Some strains of *E. coli* produce ESBLs, with the most common beta-lactamases produced being part of the TEM and CTX-M family. TEM beta-lactamases are transferred to bacteria via plasmids and include over 200 protein subtypes. These proteins act on first-generation cephalosporins and penicillin via hydrolysis, and belong to class A serine active-site hydrolases, with TEM-1 being the most common form in Gram-negative pathogens [40,41]. The majority, specifically 90%, of ampicillin resistance in *E. coli* is due to the action of TEM-1, which differs from standard beta-lactamase enzymes by a single amino acid substitution causing an alteration in the isoelectric point from a pI of 5.4 to 5.6 [41]. CTX-M beta lactamases also belong to class A, sharing the same mechanism as TEM enzymes. Their pathway involves their active-site serine attacking and producing an acyl-enzyme intermediate, which is hydrolysed. The active site is located between the two domains of class A beta-lactamases, these domains being the β and α/β domains. For the attack on the amide bond of the beta-lactam ring of the antibiotics, the oxygen belonging to the hydroxyl group of the Ser70 residue acts as a nucleophile. To avoid beta-lactamase-mediated antibiotic resistance, the production of oxyimno-cephalopsorins such as cefotaxime and ceftazidime was introduced due to their substrate binding affinity to TEM enzymes being much lower. However, single amino acid substitution mutations in TEM-1 enzymes have produced several variants that cause an increased hydrolysis of the beta-lactam ring with these named as TEM ESBLs. Some of the most frequent substitutions include E240K, G238S, M182T, A237T, R164S/H, and E104K [42]. AmpC beta-lactamases are an additional group of enzymes found in *E. coli* but also have hydrolytic activity against cefotetan and cefoxitin. The most common enzyme belonging to the AmpC enzyme found in *E. coli* is CMY-2, part of the CIT group of enzymes [43]. In *E. coli*, *AmpC* expression is significantly lower in comparison to other bacteria due to the lack of the regulator gene *ampR*. However, genes encoding plasmid-mediated AmpC beta-lactamases (*pAmpC*), such as CMY-2, are very much expressed at a much higher than average rate [43]. Class B metallo-β-lactamases are also found in several strains of this bacterium, threatening the antibiotic use of carbapenems as an alternative *E. coli* drug due to a type of β-lactamases called carbapenemases. The most recognised metallo-β-lactamase is the New Delhi metallo-β-lactamase (NDM-1). This enzyme subtype is named after its epidemiology, being frequently found in areas of India, especially New Delhi, where it is one of the main causes of community-acquired infections and diseases such as diarrheal infections. The concern of the bla_NDM-1_ gene spreading to strains in the UK, Australia, and US due to environment contamination and travel is high due to its MDR nature, resulting in apprehension for global public health [43]. Since the identification of NDM-1, a further 20 variants of the protein have been discovered, with NDM-5 being the second most predominant NDM in *E. coli*. Shen and colleagues in 2018 studied the prevalence of *E. coli* NDMs in the gut of healthy patients and livestock in China and showed that the most predominant NDM was NDM-5 rather than -1 in this region. More importantly, they showed that a select number of NDM-5 containing *E. coli* strains in the gut showed resistance to colistin due to the presence of the *mcr-1* gene. This could result in further antibiotic resistance to last-resort drugs in the future and should be urgently controlled [39,44].

#### 3.1.2. Aminoglycoside-Modifying Enzymes

Several *E. coli* strains also possess several subtypes of aminoglycoside-modifying enzymes (AMEs), responsible for their resistance against aminoglycoside antibiotics by modifying their chemical structure. Several subtypes of acetyltransferases, nucleotidyltransferases, and phosphotransferases have been identified in multiple *E. coli* strains. Aminoglycoside acetyltransferases are responsible for the addition of an acetyl group to an amine group on the aminoglycoside at positions 1, 2, 3, or 6, resulting in enzymatic inactivation of these drugs. In particular, the frequently found acetyltransferases in this microorganism are AAC(3)-II/IV and AAC(6)-Ib [45,46]. Among the nucleotidyltransferases, ANT(2″) and ANT(3″) have been found in Gram-negative bacteria including *E. coli* and are encoded by aadB and aadA genes commonly found on gene cassettes belonging to class 1 integrons. The identified phosphotransferase proteins are encoded by *strA* and *strB* genes, known as APH(6)-Ia and APH(6)-Id, respectively. These proteins are responsible for *E. coli* resistance to streptomycin, and have been shown to link to *aph*(3″)-I/II genes, which are responsible for resistance to kanamycin, identified in swine [47] and poultry in Nigeria and China [45,46,47].

#### 3.1.3. OM Remodelling

Two important genes encoding major porins known as *ompC* and *ompF* are involved in OM remodelling in *E. coli* but only one gene can be expressed at a time. These proteins limit antibiotic uptake by decreasing the quantity of the porins or an alteration of the porin charge, preventing drug entry. In this Gram-negative pathogen, these porins usually decrease in quantity or completely stop production to prevent drug uptake or produce an alternative porin [37]. When the OmpC or OmpF porins are present, the OM remodelling can occur, allowing the OM proteome to adapt and evolve in response to selective pressures such as the presence of antibiotics, e.g., aminoglycosides and beta-lactams. When *E. coli* strains are found in conditions where glucose supply is limited or in a hypo-osmotic atmosphere, the major porin expressed is OmpF, whereas when nitrogen supply is limited or in a hyper-osmotic atmosphere, OmpC is the major porin expressed. These two OM porins share several similarities in their chemical structure apart from minimal alterations in the amino acid sequence of their cell surface inter-strand loops, hence why these two proteins are referred to as OmpC/F. Several papers have shown that the presence of carbapenems and tetracyclines results in the expression of OmpF (meaning OmpC expression is not possible) [28,48]. Furthermore, when nalidixic acid is used, OmpC is expressed rather than OmpF. An *E. coli* analysis using proteomics showed that when beta-lactams or tetracyclines are used against *E. coli*, BamC and BamD, two units of the BAM complex, which forms a target site for antibiotics, are increased in expression in response to the presence of OmpC, triggering OM remodelling [28]. Furthermore, *E. coli* can activate enzymes such as the LD-transpeptidase LdtD when the OM is compromised in the presence of antibiotics, preventing cell lysis by remodelling the peptidoglycan. LPS transport from the IM to the OM in this bacillus occurs via the activity of seven key proteins called LptA-LptG, which produce a transenvelope protein bridge across the periplasm, allowing LPS movement across the cell envelope using ATP. This process is one of the main transport systems in *E. coli*, with Morè et al. in 2019 showing that when the OM is compromised, e.g., by the entry of antibiotics, LD-transpeptidases (LDTs) produce 3-3 cross links in the peptidoglycan layer. This strengthens the PG layer to prevent antibiotics reaching their target sites in the cytoplasm [49]. Due to OM remodelling being a substantial antibiotic resistance mechanism in this microorganism, Tsang et al. in 2017 studied cell wall remodelling during cytokinesis in *E. coli*, and identified a target divisome protein known as Nldp. This protein is responsible for activating enzymes AmiA, AmiB, and AmiC that cleave the bonds between the glycan strands and the stem peptides forming the PG layer and concluded that targeting Nldp as a site for antibiotics that disrupt the cell envelope could present as a new potential alternative treatment [50]. 

#### 3.1.4. Efflux Pumps

The most common antimicrobial efflux pumps found in *E. coli* belong to the RND family, with their major associated OMP being TolC. The five identified RND pumps are linked to TolC proteins and are known as MdtABC-TolC, MdtEF-TolC, AcrAB-TolC, AcrAD-TolC, and AcrEF-TolC, with pump AcrAB-TolC having the most clinical significance. This pump in particular is responsible for the efflux of several antibiotic classes including β-lactams, tetracyclines, lincosamides, fluoroquinolones, and chloramphenicol. The other four efflux pumps present in *E. coli* are expressed at a much lower level relative to AcrAB-TolC but still help with drug efflux due to their affinity for different antibiotic classes. MdtABC-TolC effluxes quinolones, MdtEF-TolC targets erythromycin, AcrAD-TolC works against β-lactams and aminoglycosides, and AcrEF-TolC effluxes tigecycline and quinolones.

Other than RND pumps, there has been a single identified ABC efflux pump found in *E. coli* named the MacAB transporter that is partially responsible for resistance against macrolides, and five identified MFS efflux pumps known as MefB, MdfA, QepA2, EmrAB-TolC, and Fsr, effective against different antibiotic classes. QepA2, MdfA, and EmrAB-TolC affect fluoroquinolones, whereas MefB and MdfA are effective against macrolides. The MFS pumps acting on tetracyclines include EmrAB-TolC and MdfA, and resistance to tetrayclines can be genetically acquired by *E. coli* by the action of plasmids carrying *tetA* and *tetB* genes [37]. Furthermore, Chetri and colleagues in 2019 performed a study on 298 non-susceptible isolates of *E. coli* and showed that the AcrAB-TolC pump also has a major role in the development of carbapenem resistance [48]. Another key efflux pump in *E. coli* is the CusBAC efflux pump, responsible for the transportation of Cu(I), Cu(II), and Ag(I). 

#### 3.1.5. Alteration of Target Sites

Resistance to quinolones and fluoroquinolones in *E. coli* mainly occurs via chromosomal and plasmid target site mutations. Fluoroquinolones target the GyrA and GyrB DNA gyrase subunits, as well as the ParC and ParE subunits of topoisomerase IV acting as a secondary target site [51]. Mutations conferring for fluroquinolone resistance have shown to be found in the QRDR region, in codons 83 and 87, between Ala67 and Gln107 residues in gyrA. A single *gyrA* mutation has shown to be sufficient for quinolone resistance, but more mutations in *gyrA/parC* are required to produce fluoroquinolone resistance. Mutations in *parC* often are located in codons 80 and 84. These mutations are common, and have previously been identified in *E. coli* strains isolated from diseased animals by Liu and colleagues in 2012 in China, and sediment samples in aquatic environments in 2015 in Sweden by Johnning and colleagues [45,52,53]. Furthermore, 16S RNA or S5/S12 ribosomal proteins targeting mutations have shown to confer resistance to aminoglycosides via the action of RNA methylases. Mutation sites confirmed to cause aminoglycoside resistance in *E. coli* are the A1408 and G1405 residue sites located on 16S ribosomal site A, resulting in significant resistance to a range of aminoglycoside drugs including gentamicin, tobramycin, amikacin, and netilmicin. This methylation is caused by ArmA methylases encoded by *armA*, horizontally disseminated through various Gram-negative ESKAPE pathogens via the composite transposon Tn1548 [45].

#### 3.1.6. Key Findings

The key findings from the literature regarding *E. coli* antibiotic resistance mechanisms conclude that beta-lactamase production in *E. coli* is a major process by which beta-lactam antibiotic resistance is mediated according to Bajaj et al., specifically highlighting concerns of the bla_NDM-1_ gene [42]. The presence of carbapenemases and ESBLs poses the highest threat from the beta-lactamase group, with these enzymes often needing a combination therapy of several antibiotics alongside BLIs, as reported by Al-Tamini et al. in 2019 and Harris and colleagues in 2015 [54,55]. This may combat the infection, but using a number of different antibiotics can further increase resistance rates. However, not all *E. coli* strains produce these enzymes, suggesting that other antibiotic resistance mechanisms might be more prominent, or instead a combination of mechanisms producing the highest AMR effect. OM remodelling of OmpF and OmpC is also another key mechanism of resistance as reported by Rosas and Lithgow in 2022 [28], but may not be sufficient in producing such substantial AMR if it were to act alone. In studies observing antibiotic resistance mechanisms in *E. coli* isolates such as work completed by Majumder and colleagues in 2021, the most observed resistance mechanisms in the isolates were the action of the AcrAB-TolC efflux pump alongside ESBL activity. Other studies support this theory that efflux pumps and beta-lactamases pose the highest AMR threat in *E. coli*, with its common dissemination through diseased meat-producing animals [45,56].

### 3.2. Acinetobacter baumannii

*A. baumannii* is an aerobic opportunistic Gram-negative pathogen, which is one of the main pathogens successfully responsible for hospital-acquired infections worldwide, accounting for up to 20% of ICU infections [57]. This bacillus is often referred to as ‘Iraqibacter’ due to its high infection rates in military soldiers and veterans serving in Iraq, with MDR *A. baumannii* spreading to hospitals worldwide, partially by cross-infection of military patients returning from war zones [58]. Due to its significant presence in hospitals, community-acquired infections are beginning to rise. Infections by this bacteria are common in immunocompromised patients that have had significantly longer hospital stays (higher than 90 days) and has shown to be isolated from the respiratory tract and oropharynx regions of patients infected with *A. baumannii*. It preferentially resides in moist areas of the body such as mucosal membranes or exposed skin from injuries [59]. Common healthcare-associated infections caused by *A. baumannii* include bacteraemia, meningitis, UTIs, lower respiratory tract infections such as pneumonia, and wound infections with their pathogenesis involving their wide range of virulence factors including phospholipases, LPS, protein secretion systems, OM porins, and iron-chelating pathways [57,60]. There are limited antimicrobial options when treating *A. baumannii* infections due to predominant strains acquiring MDR and being carbapenem-resistant. It is this that makes this microorganism one of the most serious ESKAPE pathogens, with the WHO classifying this bacterium as one of their highest priorities, urgently requiring new antibiotic development. Several studies have shown that *A. baumannii* becomes rapidly resistant to antibiotic drugs, with multiple MDR strains identified due to its high degree of genetic plasticity, allowing this Gram-negative species to have a large capacity to develop these resistance mechanisms [57,61]. The optimal treatment for *A. baumannii* used to be carbapenems such as meropenem, imipenem, and doripenem; however, due to the increasing resistance to these drugs, the current most effective treatment stands to be a combination therapy of ampicillin and sulbactam, or ampicillin and sulbactam combined with carbapenems for MDR strains [57]. The use of minocycline is also effective, but due to this, up to 20% of *A. baumannii* has acquired resistance to this drug. These minocycline-resistant strains are usually combatted with colistin, with strains resistant to colistin targeted using colistin and rifampicin, or colistin with trimethoprim–sulfamethoxazole. However, *A. baumannii*’s rapid ability to evade antibiotic effects is a serious concern, highlighting the urgency for the development of new therapeutics [8,61]. The following section will describe the mechanisms by which this antibiotic resistance occurs in *A. baumannii.*

#### 3.2.1. β-Lactamases

The inactivity of beta-lactams used to treat *A. baumannii* infections by the action of β-lactamases is a key mechanism of resistance in this bacterium, with publications in the last decade showing that horizontal gene transfer has a significant role in the acquisition of these enzymes. This is due to these bacteria possessing a natural lenience to involve exogenous DNA, with their genome commonly having large quantities of foreign DNA. It was also shown that the presence of albumin found in the blood enhances the uptake of exogenous DNA into their genome via HGT, presenting as a possible explanation for the high levels of β-lactamases found in *A. baumannii* [57,62]. *A. baumannii* produces a variety of β-lactamases including all four classes, ESBLs (class A); MBLs (class B); β-lactamases resistant to cephamycins, cephalosporins, and penicillins (class C); and OXA β-lactamases also known as OXA-type carbapenemases (class D), with the MBLs and OXA-type carbapenemases being one of the major causes of antibiotic resistance [8,63]. The main ESBLs belonging to this microorganism are part of the TEM-, CTX-, SHV-, and PER-type classes. There have been four identified classes of carbapenemases referred to as IMP-like, NDM-type, SIM-1, and VIM-like carbapenemases, with the genes that code for these proteins located on integrons being able to pass on to other bacteria via HGT. A study by Alkasaby et al. in 2017 investigated *A. baumannii* isolates in an hospital located in Egypt and found that antibiotic resistance was high for all drugs except for colistin and tigecycline, with highest resistance (>90%) to ciprofloxacin and aminoglycosides. All those isolated contained the *bla*_OXA-51_ gene, suggesting its correlation with carbapenem resistance, and 95% of isolates carried genes encoding for MBLs. These data showed the need to minimize colistin use and limit cephalosporin and carbapenem use to prevent these drugs from becoming futile, as well as the need for cautious monitoring of *A. baumannii* in healthcare settings [64]. Carbapenem resistance in *A. baumannii* is due to MBLs (class B) and OXA-type carbapenemases (class D), with identified subtypes of OXA-23-like, OXA-24-like, OXA-40-like, OXA-51-like, OXA-58-like, and OXA-143-like [63]. These class D enzymes can hydrolyse and breakdown extended spectrum carbapenems and cephalosporins, preventing their entry to the cytoplasm [8]. A study conducted by Colquhoun et al. in 2021 explored the overexpression of the beta-lactamase OXA-23, which caused the chemical and genetic prevention of the PG layer, allowing the potential for new antimicrobial sites that could kill these strains [65]. Further identified carbapenems detected in *A. baumannii* are the *K. pneumoniae* carbapenemase (KPC) β-lactamases, which are other class A serine-hydrolysing enzymes specifically active towards one of the last-resort drug classes, carbapenems. These were first identified in *K. pneumoniae* in 2001 in nosocomial strains found in North Carolina, and are rapidly spread through *K. pneumoniae* via HGT, producing MDR strains. Variants of genes encoding for the *bla*_KPC_ gene have been identified in *A. baumannii* strains, with the confirmed genes known as *bla*_KPC-2_ and *bla*_KPC-3_ found in burn patients in Brazil in 2016 [61,66]. These KPC enzymes pose as a major threat, due to their ability to hydrolyse all current FDA-approved β-lactam drugs. The drugs that show limited antimicrobial activity include clavulanate, sulbactam, and tazobactam; however, this effect is minimal and not sufficient for clinical use against resistant strains [61].

#### 3.2.2. Aminoglycoside-Modifying Enzymes

*A.baumannii* possesses several AMEs, including all subtypes of AACs, APHs, and ANTs, responsible for their resistance towards aminoglycoside antibiotics especially in MDR strains. This bacillus commonly presents with high levels of resistance to older aminoglycosides such as kanamycin and gentamycin, but recent studies have shown a newer resistance in several different countries to semi-synthetic aminoglycosides such as tobramycin, amikacin, and isepamicin. The genes encoding for AMEs are found on mobile genetic elements including transposons and plasmids and are transferred to other *A. baumannii* strains via HGT. Data from several studies have shown the predominant AMEs to be AAC(3)-I, APH(3′)-VI, and ANT(3″)-I [67,68]. The largest group of these enzymes is the APH(3′)-I class, with the majority of *A. baumannii* acquiring the aph6 gene, resulting in neomycin, kanamycin, amikacin, ribostamycin, paromomycin, and isepamicin. These enzymes cause the addition of a phosphate group on the aminoglycoside hydroxyl groups either at positions 2′, 3′, 3″, 4, 6, or 9 [69].

#### 3.2.3. Efflux Pumps

One of the most major efflux pumps described in *A. baumannii* is the AdeABC pump of the RND family, also responsible for aminoglycoside resistance alongside AMEs. As well as their role in aminoglycoside resistance, these efflux pumps are also involved in resistance to other antibiotic drugs including tigecycline, erythromycin, chloramphenicol, and tetracycline. Other than AdeABC- and RND-type pumps, other identified efflux pumps in *A. baumannii* include multidrug and toxic efflux (MATE), the major facilitator superfamily (MF), Tet(A) and (B), RND, small multidrug resistance (SMR), MacB, and ATP binding cassette (ABC). Other than the ABC pump that requires energy from ATP hydrolysis, the other pumps use proton motive power as their source of energy [70]. The AdeABC pump has a tripartite structure, the outer membrane protein known encoded by *adeC*, the multidrug transporter encoded by *adeB*, and the membrane fusion protein encoded by adeA. When effluxing antibiotics, the drug is initially captured by *adeB* in the IM of the cell envelope and transported out of the cell via *adeC*. Overall, the expression of the entire *adeABC* gene is modulated by *adeRS*, a bicomponent regulatory system composed of *adeR*, a responsive regulator protein, and a histidine kinase called AdeS that responds to environmental signals resulting in autophosphorylation [71]. It has been shown that mutations in the *adeRS* such as the Asp30 to Gly substitution can cause overexpression of this efflux pump, potentially presenting as a reason for MDR *A. baumannii* [71,72]. This regulatory system is found upstream of *adeABC*, with *adeABC* having varying expression levels for each gene encoding the three sub-proteins. Several studies have researched the expression of these genes and their relative importance, with many concluding the most crucial sub-protein of the efflux pump for its function being encoded by *adeB*. However, the study by Xu et al. in 2019 showed that *adeC* may not be as important as *adeB* function-wise but presents with increased strain resistance to all tested antibiotics with its presence resulting in a higher probability in the formation of MDR strains. Despite *adeABC* having several antibiotic substrates including fluoroquinolones, beta-lactams, tigecycline, and chloramphenicol, it provides the highest clinical resistance to aminoglycosides, with its largest effect against gentamycin and netilmicin specifically. Moreover, a further role of *adeABC* has been identified, shown to associate with AMEs to increase resistance against carbapenems, with a study in China showing *adeABC* overexpression linking to carbapenem resistance in isolated *A. baumannii* strains [71,73].

#### 3.2.4. OM Remodelling

Studies have shown that decreased expression of specific OM porins such as Omp22-33, Omp33-36, Omp37, Omp43, Omp44, Omp47, and CarO is linked to carbapenem resistance. Another OM porin referred to as OmpA has also been shown to correlate with antibiotic resistance by associating with the OXA-23 carbapenemase alongside the CarO porin [57]. OmpA is one of the most important beta-barrel OMPs, as well as a key drug resistance protein in *A. baumannii*, which has been reported to induce AMR by decreasing the rate of diffusion of negatively charged beta-lactam antibiotics. It has also been shown that OmpA has recently resulted in resistance to the last-resort antibiotic colistin in specific strains, with an isogenic mutant form of OmpA disrupting the integrity of the cell envelope, increasing sensitivity to colistin. *A. baumannii* clinical isolates have shown that a higher expression of OmpA correlates with a higher mortality rate in patients, possibly linking with efflux pumps to enhance antibiotic resistance by pushing antimicrobial drugs out of the periplasm. Furthermore, OmpA acts by binding at its C-terminal to the PG layer in *A. baumannii*, modulating membrane integrity and outer membrane vesicle (OMV) assembly [74]. Due to OmpA’s significance in *A. baumannii* virulence as well as AMR, it has been suggested in various studies that it could be a potential therapeutic target [75]. The carbapenem susceptibility porin (CarO), another key beta-barrel OM porin in *A. baumannii*, is responsible for modulating beta-lactam entry, specifically imipenem, despite its structure not being a continuous channel like other OMPs. It is classified into four types, type 1–4, with type 3 being the most common in nosocomial strains and a decrease in CarO being associated with imipenem resistance [76]. There have been a few studies reporting the association between carbapenem resistance and CarO loss in clinical strains of *A. baumannii*, with this being illustrated using the ‘porin-localized toxic inactivation’ model, which explains the action of carbapenemases such as OXA-23 associating with the periplasmic site of OMPs, i.e., CarO or OmpA, to inactive antibiotic drugs by working together to act as a selective filter [74,77].

#### 3.2.5. Alteration of Target Sites

**Figure 5:**
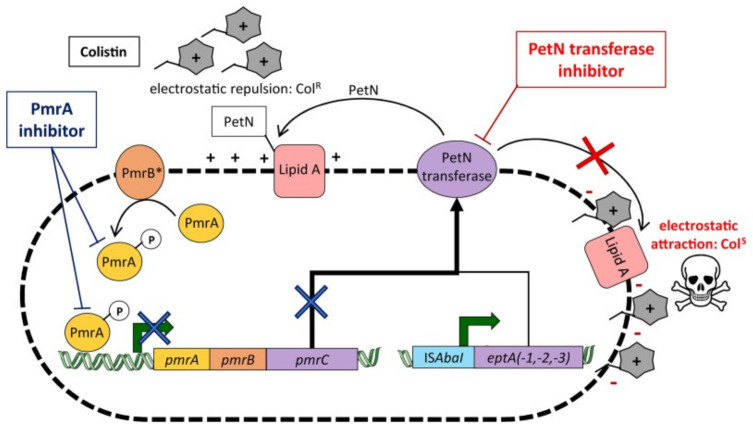
Diagram showing *A. baumannii* resistance mechanisms to colistin. The overall colistin resistance mechanisms in *A. baumannii*, with the mechanisms resulting in PetN transferase overexpression depicted, leading to colistin resistance. This is mainly attributed to overexpression of the *pmrC* gene. The diagram shows an alternative cause of PetN transferase overexpression is also due to the insertion of ISAbaI upstream of eptA(-1,-2,-3). Moreover, the use of a PmrA inhibitor shown in navy blue would inhibit the *pmrC* pathway (navy blue X), preventing PetN transferase overexpression, alongside the PetN transferase inhibitor shown in red, blocking Lipid A alterations, allowing the antimicrobial effects of colistin [80] (figure taken from [80]).

The most frequent genetic modifications are in DNA gyrases and RNA polymerases, commonly resulting in loss of affinity of imipenem to penicillin-binding protein type 2 (PBP 2) in *E. coli*. These enzymes are key in the formation of the PG layer, essential for *A. baumannii* and other Gram-negative species’ structural integrity, meaning the loss of imipenem affinity allows *A. baumannii* to evade its antimicrobial effects. Another key alteration is at the methylation of the 16S RNA ribosomal subunit found in the cytoplasm, the aminoglycoside target site by RNA methylases, with this currently conferring significant resistance to all clinical aminoglycoside drugs [78]. A study in north-eastern China showed high resistance levels to aminoglycosides in *A. baumannii* isolates, shown by determining the minimum inhibitory concentration (MIC) of 21 various antibiotic drugs, with the MIC being highest for aminoglycosides [79]. As well as these mechanisms, recent reports of colistin resistance in isolates have been detected, with a study by Trebosc et al. in 2019 studying 12 key *A. baumannii* clinical strains. In 83% of these isolates, colistin resistance was regulated by the overexpression of *pmrC*, the gene encoding for PetN transferase. PmrC is responsible for regulating the post-translational modification of Lipid A of LPS by adding phoshpoethanolamine and 4-a mino-4-deoxy-L-arabinose (L-Ara4N), increasing the charge of the cell membrane, resulting in a less negatively charged membrane, meaning polymyxin B is not able to bind as efficiently, as well as affecting the binding affinity of colistin. This overexpression was a result of mutations in PmrB, a sensor kinase that phosphorylates the response regulator PmrA (see Figure 5). Across the study, seven PmrB variants were identified in the 10 colistin-resistant strains mediated by PmrC, highlighting the diversity and range of possible mutations. In two strains, an insertion of ISAbaI also resulted in overexpression of *eptA*, *eptA-1*, and *eptA-2*, gene homologs of *pmrC* that have also been in *A. baumannii* strains but are not regulated by the PmrAB two-component regulatory system. This study also showed that International Clone 2 strains of *A. baumannii*, the most severe nosocomial *A. baumannii* strains, possessed the *eptA* genes, but the presence of this gene alone is not indicative of colistin resistance. From the findings of this study, Trebsoc et al. suggested that a potential therapeutic drug to combat *A. baumannii* resistance to colistin involves developing a direct inhibitor drug targeting the homologous PetN transferases EptA and PmrC, preventing overexpression, thus preventing any modifications to Lipid A, allowing antimicrobial action [80].

#### 3.2.6. Key Findings

From the literature, we can conclude that one of the most important mechanisms discussed is the presence of carbapenemases, especially due to the emerging spread of carbapenem-resistant *A. baumannii* strains. OXA-23, OXA-24, and OXA-58 have been reported by Palmieri and colleagues as well as Breijyeh et al. in 2020 to have a significant clinical impact, with increased production of these enzymes correlating with growing carbapenem resistance in this bacterium [8,81]. From evaluating the study by Trebosc et al. in 2019, regarding the mutations found in the *pmrA/B* two-component regulator genes and *pmrC* overexpression causing colistin resistance [80], we can conclude that the combination of *A. baumannii* strains harbouring these OXA-like carbapenemases as well as mutations leading to pmrC overexpression are particularly dangerous. Preventing the dissemination of these mutations is key to ensure colistin remains active against MDR *A. baumannii* strains. Furthermore, from the multiple resistance mechanisms *A. baumannii* has acquired, we can conclude that it must possess a much higher degree of genetic plasticity. Its susceptibility in acquiring these genes may be one of the most important mechanisms of its resistance, which suggests the need for this bacterium in particular to be regularly analysed and genetically surveyed to prevent further dissemination. Further research into this significant ability should be conducted to allow an understanding to produce more targeted therapies. Despite the important presence of efflux pumps, OM remodelling, and other beta-lactamases, we can suggest that other mechanisms discussed may be more prominent in producing *A. baumannii*’s high levels of resistance and dissemination. 

### 3.3. Klebsiella pneumoniae

*Klebsiella pneumoniae* (*K. pneumoniae*) is an encapsulated, non-motile bacterium belonging to the Enterobacteriaceae family. It is classified as an opportunistic pathogen and can also be a major cause of nosocomial infections, causing a range of diseases such as UTIs, pneumonia, bacteraemia, and liver abscesses. It is typically found on mucosal surfaces, such as the GI tract and nasopharynx of humans, or in the water or soil in the environment [82]. These bacteria have a natural resistance to penicillins and have emerged as an urgent global health threat due to MDR *K. pneumoniae* that possess ESBLS and carbapenemases that are able to evade antimicrobial killing. The carbapenem-resistant *K. pneumoniae* strains have been identified by the WHO as one of their highest priorities to develop new antimicrobial drugs [83]. Due to clinical strains of *K. pneumoniae* being able to rapidly acquire new genetic material, two different pathotypes of this bacterium have been identified, and are classified as classical *K. pneumoniae* (cKp) and hypervirulent *K. pneumoniae* (hvKp). HvKp is a growing pathotype that is commonly found in community-acquired infections, rather than in healthcare settings. HvKp is most accurately described as a highly virulent pathogen, with infection features indicative of hvKp being its ability to affect healthy individuals spanning all age groups, affecting various sites in the body. These infections are mostly found in the Asian Pacific Rim but are emerging globally, posing as a serious concern. Furthermore, hvKp possesses an increased capability in causing central nervous system diseases including endophthalmitis, which requires urgent treatment. This is due to its hypervirulent nature, with significantly increased virulence factors such as higher rates of capsule and aerobactin production, which are encoded by larger virulence plasmids combined with integrative and conjugative elements (ICEs). cKp is a much more common pathogen and is classified as part of the ESKAPE pathogens due to its large array of antibiotic resistance mechanisms, making treatment an increasing challenge [84]. Hosts for cKp are usually older patients, with some form of immunocompromise due to their weak immune system unable to fight off infections. *K. pneumoniae* accounts for 3–8% of all bacterial nosocomial infections, and 11.8% of hospital-acquired pneumonia cases globally, with 8–12% of ventilator-associated pneumonia cases also being due to *K. pneumoniae*. Immunocompromised patients, especially those suffering from diabetes, septicaemia, and alcoholism, have significantly higher mortality rates ranging from 50–100% [85]. The following section will describe the antibiotic resistance mechanisms of *K. pneumoniae*, and the current research being conducted to attempt at overcoming these. 

#### 3.3.1. β-Lactamases

*K. pneumoniae* has several β-lactamases, with class A narrow-spectrum TEM-like enzymes and SHV-1 being commonly found and conferring resistance to penicillins and cephalosporins. However, these enzymes have significantly evolved and resulted in the production of various ESBL variants conferring resistance to aztreonam and oxyimino-β-lactams. The genes encoding for these variants are located on MGEs such as plasmids, enabling their dissemination throughout different Gram-negative species via HGT. Other class A β-lactamases identified in *K. pneumoniae* as well as other Gram-negative bacteria include those part of CTX-M, GES, PER, and VEB families. Specific β-lactamases resistant to β-lactamase inhibitors have been identified in *K. pneumoniae* strains, with these plasmid-encoded enzymes known as KPC serine carbapenemases, which target carbapenem antibiotics and are able to hydrolyse nearly all β-lactams. These enzymes are associated with major outbreaks of Gram-negative species, including the outbreak by *K. pneumoniae* strain ST258. Due to these enzymes, successful combinations of drugs have been reported using several novel β-lactamase inhibitors such as cilastatin/imipenem/relebactam or meropenem/vaborbactam or ceftazidime/avibactam. However, resistance to the ceftazidime/avibactam combination has been identified in some *K. pneumoniae* strains such as ST258 carrying *bla*_KPC-3_ genes, and other variants of *bla*_KPC_ genes [12]. Further β-lactamases identified in *K. pneunomiae* include class B MBLs such as VIM- and IMP-type MBLs as well as the NDM-1 MBL, which was first identified in a highly virulent and pathogenic strain of *K. pneumoniae* in 2009 and is now found in several different Gram-negative bacteria via HGT. NDM-1 shows resistance to almost all beta-lactam antibiotics aside from aztreonam, with a total resistance level higher than 50% determined in a study by Xiang et al. in 2020 [86]. Furthermore, it has been shown that *K. pneumoniae* strains harbouring the NDM-1 enzyme also render the new semi-synthetic aminoglycoside drug plazomicin ineffective. Evidence shows that bacteria acquiring this enzyme may cause the most MDR bacteria, with *K. pneumoniae* being a major cause of NMD-1 dissemination [87]. Class D β-lactamases identified in *K. pneumoniae* include the OXA-type carbapenemases, these being OXA-48, OXA-51, OXA-181, and OXA-237, with ESBL classical oxacillinases being OXA-11 and OXA-15. These enzymes have a high level of activity towards carbapenems, and a low hydrolytic activity against clavulanic acid. These strains pose as the highest challenge in detection and treatment due to their high rates of beta-lactam-mediated resistance, with OXA-48-producing *K. pneumoniae* strains being dangerously frequent in nosocomial infections, mostly occurring in Middle Eastern and European hospitals [88].

#### 3.3.2. Aminoglycoside-Modifying Enzymes

The activity of AMEs in *K. pneumoniae* is one of the main mechanisms conferring resistance to aminoglycoside drugs. AAC(6′)-Ib was shown to be present in 98% of *K. pneumoniae* strains in a study, correlating with resistance to tobramycin and amikacin, but not gentamycin. This enzyme is assumed to be significant in most strains due to many carbapenem-resistant *K. pneumoniae* strains possessing ESBLs, with the genes encoding for ESBLs and AAC(6′)-Ib co-locating on the same plasmid [89]. Another significant AME is present in several *K. pneumoniae* strains, AAC(3)-IIa, and is more frequent in strains harbouring the *bla*_CTX-M_ gene [90].

#### 3.3.3. Efflux Pumps

The RND efflux pump superfamily in Gram-negative bacteria involves key mechanisms regulating antibiotic resistance, especially in *K. pneumoniae*. One of the main RND efflux pumps responsible for MDR strains of this bacterium is the *K. pneumoniae* acriflavine resistance B (KpAcrAB) multidrug efflux pump. AcrB is located in the IM and forms a tripartite structure by associating with the periplasmic adaptor protein KpAcrAB and the outer membrane channel protein KpTolC. Combined, this tripartite complex spans the complete bacterial cell envelope, allowing efficient antibiotic efflux via energy derived from proton motive force (PMF). To understand its precise mechanism due to its significant presence in *K. pneumoniae*, Zhang and colleagues in 2023 performed cryo-electron microscopy and computational docking in the presence of several antibiotic classes. It was shown that the KpAcrAB pump acts through a drug/proton antiporter pathway, with protons being transported into the cytoplasm to provide energy for the export of antibiotic molecules, with the amino acid residue lysine being key in proton import across the cytoplasmic membrane. It was shown that KpAcrAB can efflux a variety of antibiotic classes, including tetracyclines, chloramphenicol, quinolones, beta-lactams, macrolides, and aminoglycosides, through the presence of a multidrug binding pocket [91]. Other members of the RND efflux pump superfamily present in *K. pneumoniae* include OqxAB, EafAB, and KexD. The *oqxAB* gene is plasmid-encoded, which may pose as a risk for its dissemination through HGT to different bacterial species, resulting in AMR. This pump shows resistance to a range of antibiotic drugs, such as quinolones, tigecycline, nitrofurantoin, quinoxalines, and chloramphenicol. It is regulated by two major proteins, RarA (regulator of antibiotic resistance A), with *rarA* overexpression linked to expression of MDR *K. pneumoniae* phenotypes, and RamA, which is known as the most efficient modulator of transcription in *K. pneumoniae*. Overexpression of the *oqxAB* gene was shown to cause at least a four-fold decreased susceptibility to several different antibiotics such as fluoroquinolones and quinolones, chloramphenicol, quinoxaline drugs, and trimethoprim [92].

#### 3.3.4. OM Remodelling

Modulating the number or type of porins present in the *K. pneunomiae* OM is a major mechanism of antibiotic resistance, preventing their influx and activity on their target sites. OmpK35, OmpK36, OmpK37, OmpK38, OmpK26, and PhoE are identified as OM porins in *K. pneumoniae*, with OmpK35 and OmpK36 classified as the major porins. These two proteins have a trimeric structure formed of 16-stranded β-barrels lined with polar residues in their internal pore, shown to allow the entry of β-lactams. Loss-of-function mutations in the genes encoding for these two porins correlate with antibiotic resistance in *K. pneumoniae* strains, with ompK35 mutations being associated with increasing global β-lactam resistance [93]. These co-regulated porins (OmpK35 and OmK36) have shown to be homologs of the OmpF and OmpC major porins of *E. coli*, respectively; however, OmpK35 and OmpK36 produce channels increased in size and permeability. It has been shown that tigecycline-resistant isolates of *K. pneumoniae* have a reduced expression of ompK35, as well as this decreased expression correlating with third-generation cephalosporins and carbapenems [94]. Furthermore, mutations in OmpK36 working alongside carbapenemases are emerging in several MDR *K. pneumoniae* strains, specifically a di-amino insertion of glycine–aspartate in extracellular loop 3 of OmpK36, a region responsible for decreasing the size of the pore, preventing carbapenem influx. This emerging mechanism of OmpK36-mediated carbapenem resistance has commonly been identified in the highly virulent ST258/512 strain, spreading across South and North America as well as through Europe [95].

#### 3.3.5. Alterations of Target Sites

PBP modification and key proteins involved in PG synthesis that are acting as the target site for beta-lactam antibiotics is an important resistance mechanism. The alterations in PBPs result in a change in their chemical structure, decreasing the affinity of beta-lactams to PBPs, thus enhancing their resistance. Moreover, fluroquinolone resistance in *K. pneumoniae* commonly occurs through point mutations in the *gyrA*/*gyrB* genes encoding for the two subunits of DNA gyrase and in the *parC/parE* genes encoding for two subunits of topoisomerase IV [88]. These point mutations are usually spontaneous and are found in the amino acid sequence encoding for the 5′ quinolone-binding region of the enzymes in mainly *gyrA* and *parC*. Evidence also shows that modifications in subunit B may contribute to increased resistance, with a collection of several mutations across both enzymes resulting in the highest level of fluroquinolone resistance. Furthermore, acquisition of proteins conferring quinolone resistance such as quinolone resistance proteins (Qnr-family proteins) that protect DNA gyrase via HGT of mobile genetic elements is another mechanism by which *K. pneumoniae* become resistant to fluoroquinolones. These phenomena are known as plasmid-mediated quinolone resistance (PMQR), where Qnr proteins including QnrS, QnrA, and QnrB bind to the antibiotic target found on DNA gyrase, resulting in fluoroquinolone resistance. 16S rRNA methylation via RNA methylases is another key target site alteration conferring resistance to aminoglycosides, with 10 various methylase classes being identified globally, such as ArnA RmfH, RmfA, and NmpA, and occurs in all Gram-negative bacteria in the ESKAPE group. Genes encoding for these methylases are also found on plasmids harbouring genes encoding for other MDR factors such as *bla*_NDM_ and *bla*_OXA-23_, which increases antibiotic resistance, thus decreasing current available drugs for treatment [12]. It has also been shown that 16S rRNA methylases present in *K. pneumoniae* confer resistance to the novel semi-synthetic aminoglycoside plazomicin [89]. Furthermore, a 2017 study by Kidd and colleagues showed a key mutation in MDR strains of *K. pneumoniae*, resulting in inactivation of the *mgrB* regulatory gene. In this study, it was shown that this mutational inactivation resulted in significant LPS Lipid A remodelling modulated by PhoQR, causing *K. pneumoniae* resistance to last-resort drug polymyxins such as colistin, as well as increasing the virulence of these strains. This hypervirulence was caused by the bacterium’s new ability to avoid early activation as well as limiting the host’s defence systems alongside reducing susceptibility to human antimicrobial peptides [96].

#### 3.3.6. Key Findings

Evaluating the various resistance mechanisms found in *K. pnemoniae* is essential in understanding their role in AMR, as well as highlighting the need to comprehend the interplay between them and how this impacts treatment. ESBLs and OXA-like carbapenemases are significant beta-lactamases responsible for infections, especially in healthcare settings. The study by Kot and colleagues in 2023 determining the resistance mechanisms in 109 *K. pnemoniae* nosocomial isolates showed that more than 50% of these strains produced ESBLS and were also MDR [97], suggesting the correlation between these factors. The production of ESBLs by MDR strains is a resistance mechanism requiring attention not just in this bacterium, displaying its significant presence in MDR ESKAPE pathogens. However, geographical limitations must be considered when evaluating data sets, due to different countries having variations in healthcare systems and practise, as well as socioeconomic factors. Understanding global limitations can allow us to enhance our knowledge on the global dissemination of *K. pneumoniae*, especially in healthcare settings. Efflux pumps also play a role in AMR *K. pneumoniae*; however, only a few studies provide details on KpAcrB, such as the study conducted by Zhang and colleagues in 2023 using Cryo-EM structures [91]. If these pumps were to be studied further, their specific role in resistance may become clearer to allow enhanced efforts in combatting MDR *K. pneumoniae*. Furthermore, like *A. baumannii*, *K. pneumoniae* also possesses the significant ability to horizontally acquire resistance genes with the main mechanism known being mediated by plasmids [12]. Increasing our understanding to how this occurs so rapidly may allow the implementation of effective and suitable infection control measures, decreasing AMR rates. 

### 3.4. Pseudomonas aeruginosa

*P. aeruginosa* is an aerobic, rod-shaped Gram-negative bacteria that is ubiquitous in the environment, usually found in soil and aquatic environments, specifically in freshwater areas. It is classified as a common opportunistic pathogen, capable of causing several community-acquired infections such as folliculitis, as well as being the cause of many hospital-acquired infections such as ventilator-associated pneumonia and catheter-associated UTIs. Common reservoirs for *P. aeruginosa* in hospitals include sinks, taps, and respiratory-associated treatment equipment such as ventilators, and many more due to their optimal conditions being wet surfaces. Infections are mostly seen in immunocompromised individuals, such as those diagnosed with COPD, CF, burns, and ICU patients. Patients fitted with invasive instruments such as catheters and endotracheal tubes are more susceptible to *P. aeruginosa* infections due to this species’ significant capability in producing biofilms that are particularly challenging to recognize and can survive in harsher environments [98]. *P. aeruginosa* is one of the top-listed pathogens responsible for nosocomial infections, especially in CF patients, and is part of the ESKAPE pathogens with carbapenem-resistant *P. aeruginosa* being listed as a highest priority for the development of new antibacterial agents. MDR *P. aeruginosa* strains have a large array of virulence factors such as the production of extracellular toxins and secretion of proteases and LPS. Due to this, this bacterium possesses a significant ability to overcome antimicrobial killing via the production of intrinsic and acquired resistance mechanisms [99]. A unique feature of *P. aeruginosa* is the low permeability of its OM, resulting in high levels of intrinsic resistance and low susceptibility to antibiotics. Current recommendations of antibiotic use suggest early administration alongside a combination of anti-pseudomonal β-lactams such as carbapenem, cefepime, ceftazidime, ceftolozane/tazobactam, or piperacillin/tazobactam, in addition to a second anti-pseudomonal drug such as fluroquinolones or an aminoglycoside [100]. The following sections will discuss the resistance mechanisms that *P. aeruginosa* possesses, and the current research in understanding these mechanisms to bypass them.

#### 3.4.1. β-Lactamases

The main β-lactamase responsible for β-lactam resistance in *P. aeruginosa* is the chromosomally encoded class C β-lactamase named AmpC cephalosporinase with MDR strains having high levels of ampC expression. This enzyme allows significant resistance to cephalosporins and penicillins and can affect cefepime but has no effect on carbapenems. AmpC works alongside a transcriptional regulator known as AmpR, forming an operon, which can activate or repress expression of *ampC* by altering its conformation to regulate activity of RNA polymerase [101]. As well as AmpC, *P. aeruginosa* is able to produce class A β-lactamases of the TEM, PSE, PER, VEB, GES, BEL, and CARB subtypes with these enzymes presenting with activity towards cefepime, aztreonam, and cefpirome but not towards ceftazidime and carbapenems. *P. aeruginosa* is able to produce class D β-lactamases as well, referred to as oxacillinases or OXA-type enzymes. Most of the genes encoding for these OXA-type enzymes are incorporated into the bacterial genome via HGT, aside from the intrinsic OXA-type enzyme OXA-50. OXA51, OXA-2, and OXA-10 (classical OXA-type enzymes) found in *P. aeruginosa* cause resistance towards ureidopenicillins and carboxypenicillins, with beta-lactamase inhibitors not having much effect on these enzymes. Extended-spectrum OXA-type enzymes found in this bacterium include variants of OXA-2 and OXA-10, caused by point mutations, and are found on MGEs such as integrons and plasmids, which increase the probability of their circulation around different Gram-negative bacteria. These enzymes present with an enhanced hydrolytic activity against cefepime, ceftazidime, and aztreonam, conferring resistance to most beta-lactamase inhibitors. Furthermore, various carbapenemases have also been identified in *P. aeruginosa*, part of the class A KPC group or KES-2 types, as well as class B MBLs. KPC enzymes are acquired by *P. aeruginosa* via HGT from Enterobacteriaceae species, whereas GES-2 is a variant of the ESBL GES-1 caused by a point mutation. The main carbapenemases found in *P. aeruginosa* belong to the MBL class and can be divided into five classes: VIM, IMP, SPM, NDM, and GIM. Only one type of NDM, SPM, and GIM carbapenemases has been found (NDM-1, SPM-1, GIM-1), whereas for VIM and IMP enzymes, multiple variants have been identified. These carbapenemases are also found on MGEs like the variants of OXA-2 and OXA-10, also resulting in a higher probability of their dissemination. These MBLs confer high rates of resistance to all current β-lactams aside from the monobactam aztreonam, as well as carbapenemases, as well as conferring resistance to beta-lactamase inhibitors [100].

#### 3.4.2. Aminoglycoside-Modifying Enzymes

AMEs are key enzymes resulting in aminoglycoside resistance, with genes encoding these enzymes found on MGEs such as plasmids. The AME most observed in *P. aeruginosa* belongs to the ANT and AAC classes, with these enzymes being able to alter most aminoglycosides due to this bacterium being able to carry multiple AMEs that can act on a wide array of different aminoglycoside drugs. The aminoglycoside that provides the highest antimicrobial activity against these enzymes due to their structure not being an effective substrate is amikacin [100].

#### 3.4.3. Outer Membrane Remodelling

OM remodelling is a key antibiotic resistance mechanism of *P. aeruginosa*, acting by altering the number or type or porins in the OM, dictating what extracellular substances are influxed into the cell. There is a wide array of porins present in the OM, ranging from specific porins (OprB, OprD, OprE, OprO, OprP), non-specific porins (OprF), gated porins (OprH and OprC), to efflux porins (OprM, OprN, and OprJ). These porins have different functions, restricting in turn antibiotic entry and increasing rates of resistance. OprH directly associates with LPS, which enhances OM stability and integrity, modulating antibiotic resistance, whereas OprM, OprN, and OprJ (efflux porins) work alongside active efflux pumps to decrease the antibiotic concentration within the cell by increasing efflux [99]. Regarding its overexpression, OprF is a key porin in antibiotic resistance, aiding *P. aeruginosa* in the formation of thick biofilms that prevent antibiotic entry. Multiple studies have shown the role of OprF linked to outer membrane vesicle (OMV) formation and biogenesis, with these OMVs providing a key role in Gram-negative bacteria functions such as increasing bacterial survival linked to stress, translocation of virulence factors, bacterial adhesion, iron and nutrient attainment, antibiotic resistance transfer, and biofilm formation [102]. OMVs also play a role in the transportation of antibiotic resistance molecules or enzymes, including β-lactamases, increasing rates of resistance [99]. Biofilm formation is a key antibiotic resistance mechanism especially in *P. aeruginosa*, with these structures being defined as a bacteria aggregate surrounded by a self-generated complex composed of extracellular polymeric substances (EPSs) of various proteins attached to the cell surface. This matrix is composed of various polysaccharides, proteins, extracellular DNA (eDNA), and lipids, allowing *P. aeruginosa* to withstand harsher conditions such as the presence of antibiotics, aids direct cell-to-cell contact for conjugation, and involves an influx of nutrients for cell survival [103]. Mechanisms by which biofilm contributes to AMR include the release of antibiotic-modifying enzymes such as beta-lactamases and AMEs, increased efflux, enhanced HGT, aggregation of filamentous bacteriophages, and associations between various Gram-negative species with different biofilms [12].

#### 3.4.4. Efflux Pumps

There have been four major efflux pumps found in *P. aeruginosa*, known as MexCD-OprJ, MexAB-OprM, MexXY, and MexEF-OprN. These efflux pumps belong to the RND efflux pump family, meaning they too have a tripartite structure. This is composed of a periplasmic membrane fusion protein, in these pumps being MexC, MexA, MexX, and MexE, respectively; a resistance–nodulation–cell-division transporter unit, MexD, MexB, MexY, and MexF, respectively; and thirdly, an outer-membrane factor forming the channel, such as OprJ, OprM, and OprN, respectively. The MexAB-OprM is the largest contributor to AMR in *P. aeruginosa* out of the efflux pumps listed. The expression of this operon is regulated via the action of repressor genes being *mexR*, *nalC*, and *nalD* regulating the pump’s activity. Antibiotic use in *P. aeruginosa* has shown to cause an overexpression of this pump, by spontaneous mutations in the repressor genes such as nonsense frameshifts and substitutions resulting in translational errors, and disruption via insertion sequences altering the overall molecular structure of repressors. The MexAB-OprM is responsible for the efflux of multiple antibiotic classes such as macrolides, quinolones, the majority of β-lactams, tetracyclines, chloramphenicol, and lincomycin. MexAB-OprM overexpression is also correlated with resistance to most antibiotics used to treat *P. aeruginosa*, apart from colistin, with carbapenem-resistant *P. aeruginosa* strains producing carbapenemases showing a significant degree of MexAB-OprM overexpression. Furthermore, overexpression of the gene encoding for this pump alongside AmpC activity has shown to produce a synergistic effect in antibiotic resistance, aside from the use of imipenem, imipenem/relebactam, and ceftolazone/tazobactam. MexXY has been shown to associate with the outer membrane factor OprM from MexAB-OprM to produce a multidrug efflux pump, due to this pump not possessing a gene encoding for its own outer membrane factor. MexXY correlates with aminoglycoside-associated resistance by working in synergy with AMEs, where *mexXY* overexpression is seen in strains particularly expressing AMEs. As well as bearing resistance to aminoglycosides, it has also been shown that MexXY is involved in resistance to other antipseudomonal drugs similar to MexAB-OprM. Both MexCD-OprJ and MexEF-OprN are expressed at a lower level than the other two pumps discussed, but are still correlated with resistance to fluroquinolones and chloramphenicol/quinolones, respectively. Despite studies producing many candidates for agents acting as efflux inhibitors targeted towards *P. aeruginosa*, the wide range of efflux pumps with various physiochemical features, efflux constants, and substrate specificities poses as a serious challenge in producing a suitable drug to combat MDR strains due to a lack of current understanding towards these variables [33,100].

#### 3.4.5. Alteration of Target Sites

Target site alteration is a major mechanism of antibiotic resistance, with a range of modifications made in the bacterium. *P. aeruginosa* resistance against aminoglycosides occurs via the methylation of 16S ribosomal RNA (rRNA) by RNA methylases, against fluoroquinolones via topoisomerase IV and DNA gyrase mutations, against beta-lactams via the adjustment of penicillin-binding proteins (PBPs), and against polymyxins via LPS modulation and alteration. The catalytic methylation of 16S rRNA in *P. aeruginosa* occurs via methylases encoded by genes found on MGEs such as transposons and plasmids, with these enzymes known as RmtA and RmtB methylases, whereas beta-lactam- and quinolone-associated resistance occurs via target site alteration by proteins whose genes are encoded on the bacterial chromosome. Topoisomerase IV (ParC) and DNA gyrase (GyrA) are important enzymes linked to cell function and survival due to their role in DNA replication. When these enzymes are altered, *P. aeruginosa* becomes resistant to the majority of the quinolone family [100]. More specifically, alterations in DNA gyrase are mainly found in the *gyrA/gyrB* genes located in the quinolone-resistant-determinative region (QRDR) motif, which is part of the active site in the DNA gyrase enzyme. The alteration of the A and B subunits due to the mutations in the amino acid sequence encoding the QRDR motif results in a modified enzyme, decreasing its binding affinity to quinolones. The mutations occurring in topoisomerase IV are classified as point mutations in the amino acid sequence encoding for *parC* and *parE* genes, which make up the ParC and ParE enzymatic subunits, conferring resistance to fluoroquinolones. Moreover, further mechanisms producing fluoroquinolone-associated resistance in *P. aeruginosa* are mutations in the genes *nalB*, *nfxB*, and *nfxC*, resulting in their overexpression, leading to overactive MexAB-OprM, MexCD-OprJ, and MexEF-OprN efflux pumps [104]. Alteration of PBPs in *P. aeruginosa* mainly occurs in PBP3, or is an overexpression of genes encoding for PBP3, which results in beta-lactam antibiotics not being able to bind to their target site and has a key role in beta-lactam-mediated resistance. Polymyxin-mediated resistance in *P. aeruginosa* is observed via alterations to the target site, which is the bacterial LPS found in the OM. This modification occurs in the Lipid A region of LPS, resulting in a decrease in the LPS negative charge by the attachment of L-Ara4N and PetN regulated by their relative operons arnBCADTEF and pmrCAB. These operons are mediated by two-component regulatory systems PmrA/PmrB and PhoP/PhoQ, as well as ParR/ParS, CprR/CprS, and ColR/ColS. The main mechanism in *P. aeruginosa* for polymyxin resistance occurs via the arnBCADTEF operon involved in L-Ara4N addition to Lipid A. Furthermore, the overexpression of OMP OprH also results in polymyxin resistance, as OprH binds to divalent cation regions of LPS, preventing polymyxin binding [100].

#### 3.4.6. Key Findings

When evaluating the literature regarding *P. aeruginosa* antibiotic resistance mechanisms, the extensive formation of biofilms in this bacterium seems to be unique, with this being able to enhance the horizontal dissemination of resistant genes as well as increasing the release of beta-lactamase enzymes. Studies have shown that it is this key feature of *P. aeruginosa* that allows its long-term persistence and protection from bacteria, such as the study by Thi and colleagues in 2020 investigating this. Several others have reported the significance of this resistance mechanism [8,105,106], with all studies pointing towards treatment that could combat this *P. aeruginosa*-specific biofilm, allowing a more narrow-spectrum treatment to decrease *P. aeruginosa* resistance levels to the furthest extent. Furthermore, the high numbers of efflux pumps present in this bacterium present as an efficient target, suggesting the potential development of efflux pump inhibitors as adjuvants due to several research studies published regarding these efflux pumps at a molecular level [33,100]. Despite the identification of several efflux pumps in *P. aeruginosa* with more published data on this resistance mechanism, it also provides limitations due to the extent of efflux pump diversity present, which requires specifically structured or charged substrates. Cost and design present as issues due to this fact, but also the potential for further resistance if the designed EPIs are broad-spectrum rather than narrow-spectrum. A summary of key resistance mechanisms that have been reported in *E. coli*, *A. baumannii*, *K. pneumonia*, and *P. aeruginosa* is presented in Table 1 below. 

## 4. Future Directions and Conclusions

MDR Gram-negative bacteria pose a grave and concerning threat to public health, particularly with the emergence and spread of the plasmid-encoded mobile genetic element (MGE) mcr-1. This MGE encodes for colistin resistance and is now found among the ESKAPE pathogens, rendering the last-resort drug colistin ineffective [107]. The dissemination of this gene highlights the urgent need for future research and development of new therapies.

Although numerous strategies have been employed to combat MDR superbugs, only a few beta-lactamase inhibitor (BLI) antibiotic adjuvants have been able to produce a significant clinical impact. The use of antibiotic adjuvants, especially BLIs, offers several advantages in limiting antimicrobial resistance (AMR) rates, including prolonging the effectiveness of existing drugs. However, their continued use may eventually lead to the horizontal dissemination of resistance genes among the ESKAPE pathogens.

One potential avenue that deserves further exploration is the development of narrow-spectrum drugs targeting specific mechanisms in particular bacteria. Recent advancements in artificial intelligence (AI) have demonstrated their potential for significantly reducing AMR rates. For instance, AI-guided learning has facilitated the development of a novel antibiotic targeting lipoprotein trafficking in MDR *A. baumannii*. This antibiotic selectively kills *A. baumannii* without affecting other MDR superbugs, offering a narrow-spectrum activity that prevents the spread of resistance genes to other species [108]. This species-specific approach not only minimizes the negative impact on the gut microbiome by preventing dysbiosis but also improves the efficacy of treatment.

The ability of AI to efficiently process and analyse large data sets presents researchers with opportunities to gain a better understanding of AMR patterns and uncover hidden insights. It enables the identification of resistance genes that may have been missed using traditional methods. Moreover, AI holds the potential to predict future resistance genes, allowing for proactive measures rather than reactive damage control. The applications of AI in the fight against AMR are vast, including reducing the cost and time required for developing new antimicrobial drugs. Additionally, AI can be harnessed as a diagnostic and treatment tool, preventing the inappropriate prescribing and exploitation of antibiotics.

Additional research within the field of antimicrobial resistance must aim to discover compounds with more suitable toxicological profiles due to many identified compounds being unable to continue in development due to this issue. However, some advances in this field are promising, including recent polymyxin B peptides, SPR741, in Phase 1 clinical trials, which have a decreased toxicity towards the kidneys, and the continued alteration of potential EPI compounds [109,110]. The use of machine-based learning and AI to provide suggestions for the improvement in toxicological profiles of antibacterial agents may aid this future direction, allowing for the exploration of structural alterations that could be made to existing drugs.

Improvements in antibiotic stewardship programmes should be implemented to help fight the high antibiotic resistance rates, especially limiting the use of carbapenems particularly in nosocomial settings [111]. Teamwork between experts in infection and primary care physicians to observe algorithms in treatment must be made, to ensure efforts in decreasing inappropriate antibiotic use as well as researching novel drug ideas are not made futile. With the WHO prioritising the ESKAPE pathogens, we must continue to observe and record infection rates of these pathogens, potentially using AI to help regularly measure these.

Overall, the WHO classification of priority pathogens provides an efficient framework for the research and development of new antimicrobial drugs to limit antibiotic resistance rates in MDR Gram-negative bacteria. The development of these future drugs is entirely dependent upon and requires a significant increase in global efforts from governments to combat these superbugs, alongside an enhanced education and implementation of rules regarding antibiotic prescription. Local knowledge concerning AMR causes and the urgent need to prevent its exponential increase is not sufficient, highlighting the need for antibiotic stewardship programmes with the potential help of AI to increase programme speed and efficiency. With the recent promising development in the use of machine-based learning and AI in antibiotic development, the future for targeting other Gram-negative ESKAPE pathogens is hopeful. However, a deeper understanding into the specific antibiotic resistance mechanisms at a molecular level is required for each species to allow their exploitation, suggesting another aspect of research requiring increased efforts to combat MDR Gram-negative pathogens.

## Figures and Tables

**Table 1:** Summary of Antibiotic Resistance Mechanisms in *E. coli*, *A. baumannii*, *K. pneumoniae*, and *P. aeruginosa*.

	Beta-Lactamases	AMEs	Efflux Pumps	Altered Target Sites	OM Remodelling
*E. coli*	TEM-1 CTX-M CMY-2 NDM-1, -5	AAC(3)-II/IV, AAC(6)-Ib ANT(2″), ANT(3″) APH(6)-Ia, APH(6)-Id	MdtABC-TolC AcrAB-TolC AcrEF-TolC MdtABC-TolC MdtEF-TolC AcrAD-TolC	*gyrA* (codons 83 and 87) *parC* (codons 80 and 84) mutations 16 rRNA mutations S5/S12 ribosomal protein mutations	OmpC, OmpF BamC, BamD LD-transpeptidase AmiB, AmiC
*A. baumannii*	OXA-23, -24, -40, -51, -58, -143. KPC-2, -3 TEM CTX SHV PER IMP NDM SIM-1 VIM	AAC(3)-1 APH(3′)-VI ANT(3″)-1	AdeABC MATE MF Tet(A) and (B) SMR MacB ABC	Methylation of 16S rRNA Mutations in DNA gyrases and RNA polymerases → decreases affinity of imipenem to PBP2. Mutations in *pmrB*→overexpression of *pmrC* Overexpression of *eptA*, *eptA-1*, *eptA-2*	Omp22–23, 33–36, 37, 44, 47 CarO OmpA
*K. pneumoniae*	TEM SHV-1 CTX-M GES PER VEB KPC-3 and other KPC-like enzymes NDM-1 OXA-48, -51, -181, -237, -11, -15	AAC(6′)-Ib, AAC(3)-IIa	KpAcrAB OqxAB EafAB KexD	PBP *gyrA* and *parC* mutations DNA gyrase binding proteins—QnrS, QnrA, QnrB Methylation of 16S rRNA by ArnA, RmfH, RmfA, NmpA Inactivation of *mgrB* → Lipid A remodelling	OmpK36, K38, K26 PhoE
*P. aeruginosa*	AmpC TEM PSE PER VEB GES-1, -2 BEL CARB OXA-50, -1, -2, -10 KPC KES-2 NDM-1 SPM-1 GIM-1 VIM IMP	ANT AAC	MexCD-OprJ MexAB-OprM MexXY MexEF-OprN	Methylation of 16s rRNA PBP LPS *gyrA* and *parC* mutations Mutations in *nalB*, *nfxB*, *nfxC* → MexAB-OprM, MexCDOprJ, MexEF-OprN hyperactivity PBP3 Lipid A *oprH* overexpression	OprB, D, E, O, P, F, H, C, M, N, J

## Data Availability

Not applicable.
